# Multiple hollow-core anti-resonant fiber as a supermodal fiber interferometer

**DOI:** 10.1038/s41598-019-45771-2

**Published:** 2019-06-27

**Authors:** Xiaosheng Huang, Jichao Zang, Seongwoo Yoo

**Affiliations:** School of Electrical and Electronics Engineering, The Photonics Institute, Nanyang Technological University, Singapore, 639798 Singapore

**Keywords:** Imaging and sensing, Photonic devices

## Abstract

Hollow-core anti-resonant fiber technology has made a rapid progress in low loss broadband transmission, enabled by its much reduced light-material overlap. This unique characteristic has driven emerging of new applications spanning from extreme wavelength generation to beam delivery. The successful demonstrations appear to suggest progression of the technology toward device level development and all-fiberized systems. We investigate this opportunity and report an in-fiber interferometer built in a dual hollow-core anti-resonant fiber. By placing multiple air cores in a single fiber, coherently interacting transverse modes are excited, which becomes a basis of an interferometer. We use this hollow core based inherent supermodal interaction to demonstrate highly sensitive in-fiber interferometer. Unique combination of the air guidance and the supermodal interaction offers robust, simple yet highly sensitive interferometer with suppressed temperature cross-talk that has been an enduring problem in fiber strain sensing applications. The in-fiber interferometer is further investigated as a sensing element for pressure measurement based on an interferometric phase change upon external strain. The interferometer features 39.3 *nm*/*MPa* of ultrahigh sensitivity with 0.14 *KPa*/°*C* of negligible gas pressure temperature crosstalk. The performance, which is much improved from prior fiber sensors, testifies advances of hollow core fiber technology toward a device level.

## Introduction

Since its first appearance in 2002^1^, hollow-core anti-resonant fiber (HAF) has attracted a significant interest for its multiple and broad transmission bands. With nearly two decades’ research efforts, focused on a negative core curvature, a non-touching core boundary, simplifying the fiber structure to one cladding ring, and further optimizing the design parameters^2–5^, a very low transmission loss of 7.7 *db/km* became achievable at 750 *nm*^5^, which is a remarkable progress from the early HAF having a few 1000 s *db/km* of a background loss^1^. The advances in fabrication and design encourage the technology to investigate anti-resonant fiber based components and devices. For example, a dual hollow-core anti-resonant fiber (DHAF) was reported as an air core air gap fiber coupler that shows the potential for interesting applications such as ultrafast laser power coupling and mid-infrared or ultraviolet light coupling where current solid core fiber couplers are unable to offer^6^.

Fiber sensors see vast applications in areas of biomedicine^7–9^, automotive industries^10,11^, and environmental monitoring^12–14^ due to their benefits of a compact size, multiplexing capability, long interaction length, easy signal detection, and an immunity to electromagnetic interference. Among the fiber sensors, a fiber modal interferometer, becomes a popular choice thanks to their high sensitivity. The modal interferometers hinge on interaction among guided modes in a single fiber^15,16^ or between two adjacent fibers^17–19^. The generated interference phase changes under measurand interaction, which is ascribed to characteristics of a sensor. However, the modal interferometers experience unwanted temperature and polarization dependence^16,17^, which hinders the widespread uptake of fiber sensors in field applications. Such undesired temperature and polarization sensitivity is greatly mitigated in HAFs. Airy guiding modes of HAF show ultralow overlap with thermal sensitive silica cladding^5,20^, thus being insensitive to temperature variation^13^, and negligible overlap between airy guiding modes and silica walls offers low birefringence^21,22^. On the other hand, a large air-filling fraction renders HAF extremely sensitive to mechanical force such as compression, bend, and strain^6,23,24^. Therefore, combination of HAF and modal interferometer represents a promising perspective in sensing application.

In this work, we use a DHAF to investigate the opportunity of advancing the fiber interferometric sensors in a hollow core fiber platform. The DHAF acts as a coupler and exhibits intrinsic supermodal interaction that serves as a basis of an interferometric sensor. Taking advantage of the supermodal interference in the dual core fiber and the air guiding of HAFs, DHAFs promise novel characteristics of high sensitivity with much suppressed temperature and polarization dependence. To the best of our knowledge, this is the first investigation of applying supermodal interaction in a multiple hollow cores fiber. We note that a single hollow core fiber has been investigated for various sensing applications for strain^25–27^, pharmaceutical detection^28^, fluorescence^29^, ring resonator for rotation sensing^30^, magnetic field sensor^31^, surface plasmon resonance sensor^32^, fiber inclinometer^33^, and gas phase chemiluminescent detection^34^, to name a few, which indicates strong interest of utilizing the hollow core structure for sensors. We extend this interest toward an in-fiber interferometer built on a multiple hollow core fiber.

## Results

### Hollow-core supermodal interferometers

**Figure 1:**
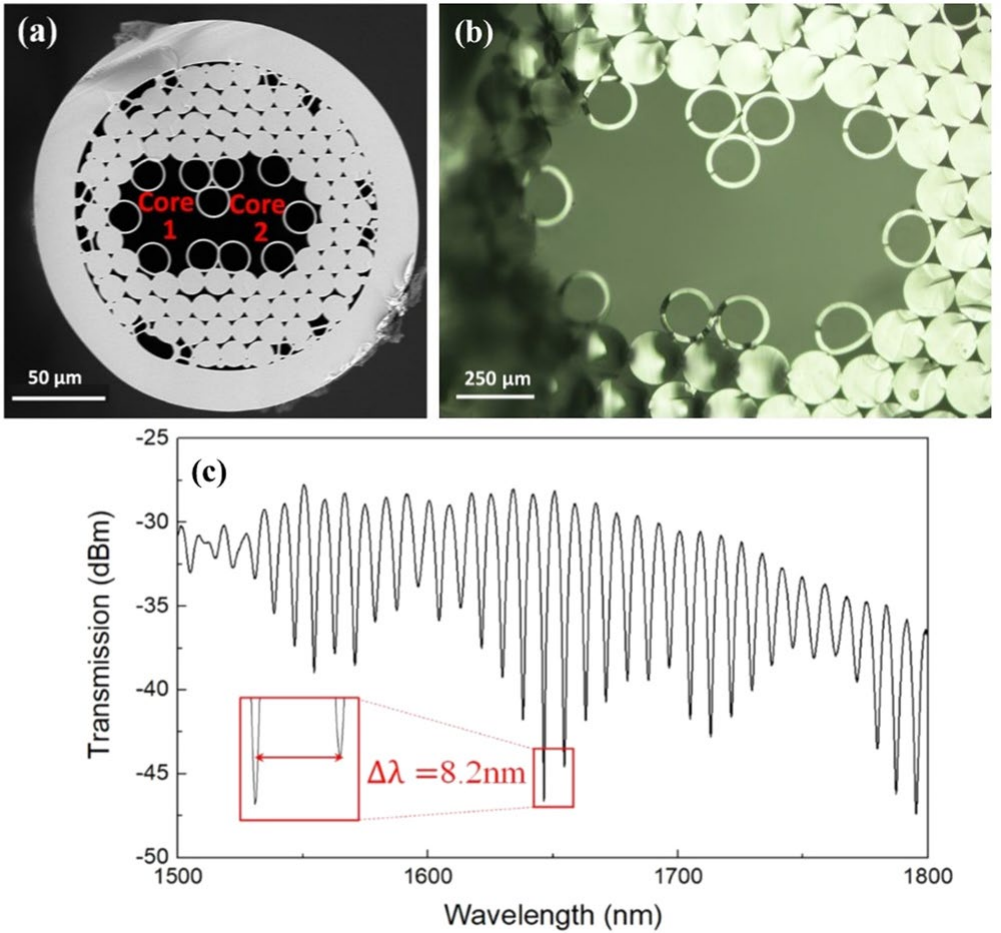
(**a**) Cross sectional view of a fabricated DHAF. (**b**) Cross sectional view of a fabricated cane. (**c**) Transmission spectrum of a 38 cm DHAF shows modal interference induced oscillatory pattern.

A DHAF structure is represented in Fig. 1(a). The fiber is composed of two hollow cores which are connected through an air channel. The single layer of capillaries introduces strong “inhibited coupling”^5^ and tightly confine the guiding modes inside the hollow-core areas. Each core works as an anti-resonant waveguide supporting transmission band in 1350–2000 *nm*, which corresponds to a normalized frequency interval from 1.88 to 1.27. The normalized frequency is defined as $F=2t\sqrt{{n}^{2}-1}/\lambda ,$ where *n* is the refractive index of cladding material (1.45), *t* is the wall thickness (as defined in Fig. S1(a) in the supplementary document) and *λ* is the wavelength. When *F* closes to an integer, there is a high loss region, and a low loss transmission band exists between every adjacent high loss regions^20^. The air channel bridging the two cores enables evanescence field interaction and supermode formation. Consequently, power coupling between the air cores is enabled^6^. We also note that modal dispersion can be included in the analysis when required^35^. Figure 1(c) shows a transmission spectrum from the output end of Core 1 when a supercontinuum source is launched into the same core and construct a white light interferometer. The transmitted spectrum was measured by an optical spectrum analyzer (OSA) at 0.04 *nm* resolution. Clearly, the anticipated periodic oscillatory pattern is generated with a slow modulated amplitude profile in wavelength regions from 1500 to 1800 *nm*. The wavelength spacing between *m*^*th*^ and (*m*−1)^*th*^ fringe dips can be approximated as:

1 $${\rm{\Delta }}{\lambda }_{m}=\frac{4{\rm{\Delta }}{n}_{eff}L}{(2m+1)(2m-1)}\approx \frac{{{\lambda }_{m}}^{2}}{{\rm{\Delta }}{n}_{eff}L}$$

where *L* is the fiber length, *λ*_*m*_ is the wavelength of *m*^*th*^ interference minima, and Δ*n*_*eff*_ is the effective refractive index (*n*_*eff*_) difference between supermodes^15,36^.

Our simulation shows that the Δ*n*_*eff*_ between the first two lowest supermodes is 8.5 × 10^−4^ at 1650 *nm*, and this agrees with the observed free spectral range in the spectrum. The slowly varying envelope is attributed to interference between symmetric and antisymmetric supermodes whose Δ*n*_*eff*_ is much smaller (please, see section 1 of the supplementary document for details). The wavelength of the *m*^*th*^ interference dip can be written as:

2 $${\lambda }_{m}=\frac{2{\rm{\Delta }}{n}_{eff}L}{2m+1}$$

As a result, any change of Δ*n*_*eff*_ by measurand interaction can shift the wavelength of a fringe dip.

**Figure 2:**
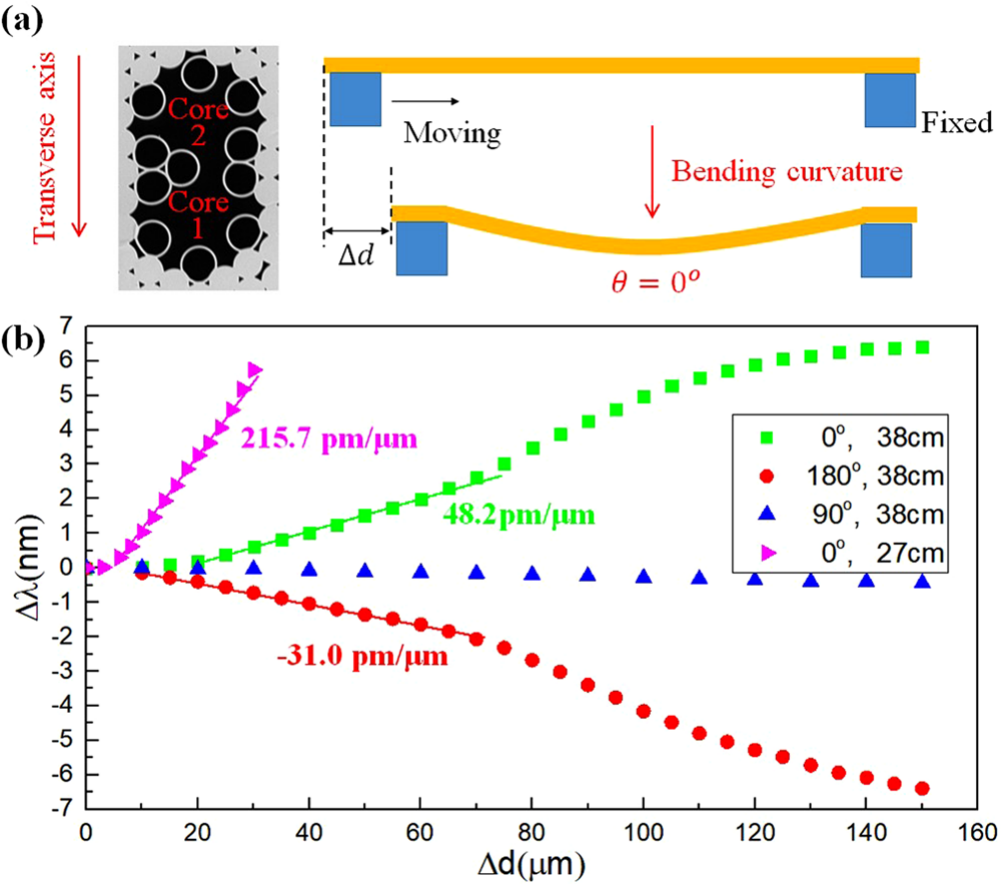
(**a**) Schematics of interferometric fiber device, *θ* is the angle between the bending plane and the transverse axis. (**b**) Performance of hollow core fiber interferometer with a response to curvature. Interference fringe shifts to opposite spectral directions because of structural distortion by the bending.

Bending is known to induce structural deformation of the hollow core fiber^23^, which will obviously influence Δ*n*_*eff*_, thus a fringe phase. We implement the bending induced Δ*n*_*eff*_ change by applying axial displacement of the DHAF. As illustrated in Fig. 2(a), one end of the DHAF is mounted on a fixed stage while the other end is placed on a translational stage to allow axial movement. The applied axial displacement, Δ*d*, renders fiber bending where the bending curvature is proportional to the displacement. In addition, we define a bending orientation by introducing an angle, *θ*, measured between a transverse axis along the two air cores and a bending plane. The transverse axis is illustrated in Fig. 2(a). When the angle, *θ*, becomes 0° or 180°, the transverse axis is within the bending plane. More specifically, Core 1 as marked in Fig. 2(a) becomes an outer core of the curvature when the angle, *θ*, becomes 0° while Core 1 becomes an inner core of the curvature when the angle, *θ*, becomes 180°. On the other hand, the transverse axis becomes out of the bending plane at *θ* = 90°. Relationship between wavelength shift of fringe dips, Δ*λ*, and axial displacement, Δ*d*, under different bending orientations, *θ*, is studied in Fig. 2(b).

The Core 1 serves as an input as well as an output ends. When the transverse axis is in the bending plane (*θ* = 0° or 180°), clear fringe shifts are observed but in opposite directions. Bending at *θ* = 0° induces red-shift while bending at *θ* = 180° shifts the fringe dips to a shorter wavelength. Despite the opposite shifts, their shift rate, i.e. Δ*λ/*Δ*d* is comparable. We also found that out of the axis bending (*θ* = 90°) does not influence the interference phase as presented in Fig. 2(b). The in-plane bending creates strain distribution across Core 1 and Core 2, accounting for the shift as well as the shift direction. Under the bending orientation with *θ* = 0°, Core 1 is subject to longitudinal tensile stress, and compressive stress at *θ* = 180°^37^.

Thus, it is postulated that the induced stress result in deformation of core structure, leading to the bending orientation dependent wavelength shift (refer to section 2 of the supplementary document for detailed discussions). If bending orientation needs control, the fiber outer-cladding can be double D-shaped. The double D-shaped fiber has longer and shorter cross-sectional axes, and the fiber will naturally bend along the shorter axis as the fiber is thinner along the shorter axis^38^. Hence, bending orientation can be controlled. The controlled fringe shift direction becomes a unique feature of this DHAF device, adding a new dimension of interrogation in a strain sensor. Furthermore, the interference phase changes more sharply in a shorter DHAF as given by the magenta line in Fig. 2(b). It is inferred that the same amount of the axial displacement, Δ*d*, is translated to larger stress in a shorter fiber. The sensitivity is improved by more than 4 times when the fiber interferometer is shortened to 27 *cm* from 38 *cm*.

**Figure 3:**
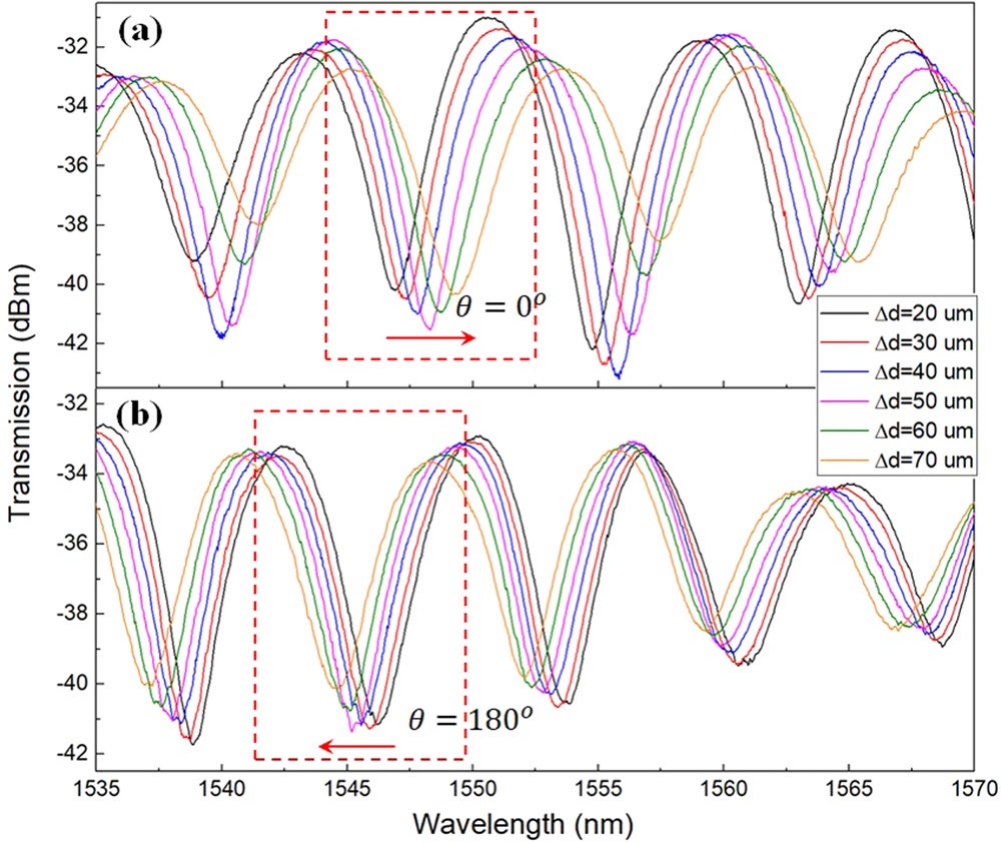
Interference fringes from transmitted light at bending orientation of (**a**) *θ* = 0°; and (**b**) *θ* = 180°. Fiber length is 38 *cm* in both cases.

Figure 3(a,b) show the transmission spectra collected from Core 1 under the axial displacement in a range of 20 to 70 *μm*. The bending orientation dependence is clearly shown in that the fringe dips undergo red shift at *θ* = 0° and blue shift at *θ* = 180°. Both shifts exhibit similar shift rate. The dip at wavelengths of 1546.87 *nm* and 1546.24 *nm* were chosen as a reference to decide the sensitivity for the bending orientations of *θ* = 0° and *θ* = 180° respectively. It should be noted that the equal spacing between every two adjacent dips confirms the linear dependence of Δ*λ* with Δ*d*.

### Demonstration of super-sensitive gas pressure sensor with DHAF interferometer

**Figure 4:**
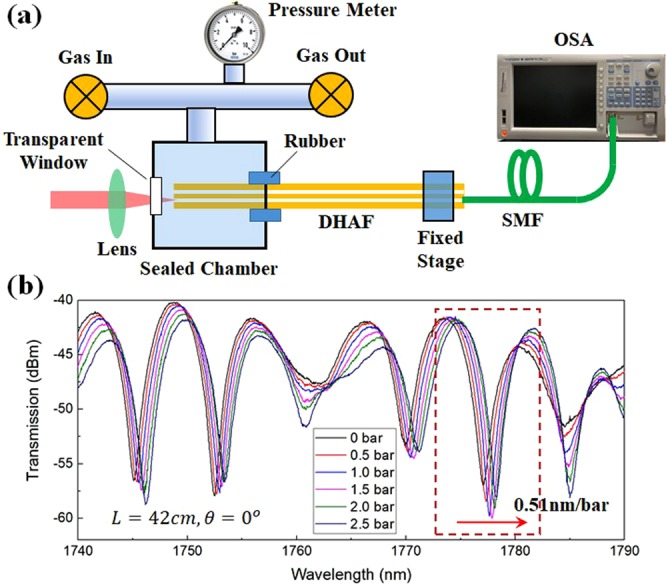
(**a**) Schematic diagram of experiment setup for hollow core fiber interferometric gas pressure sensing. SMF: single mode fiber; OSA: optical spectrum analyzer. (**b**) Spectral response of the interferometer with increasing gas pressure, *θ* = 0° (See Fig. 2(a)), *L* = *cm*.

The linear and sensitive change of Δ*λ* under curvature is desired characteristics for strain sensors. The DHAF can be attached to an elastic material which will be compressed under external mechanical force. As an elastic material responds linearly to applied force^39^, the attached DHAF will produce linear interference phase change. This motivates us to develop the DHAF interferometer into an optical fiber based gas pressure sensor, which is one of the most important optical fiber devices in industrial and environmental safety monitoring, and biomedical applications^40–42^. The schematic diagram of the in-fiber interferometric sensor is depicted in Fig. 4(a). One end of a DHAF is attached to an elastic rubber ring in a gas chamber, whilst the other end is mounted on a fixed stage. As the gas chamber is pressurized, the rubber is gradually compressed, resulting in axial displacement of the DHAF. The bending orientation is kept at *θ* = 0°. Figure 4(b) evidences the anticipated linear fringe shift toward a longer wavelength with the increasing gas pressure. The gas pressure sensitivity of the 42 *cm* DHAF interferometer is measured as 0.51 *nm*/*bar*, and this sensitivity is subject to the adopted elastic material characteristics and the fiber length.

**Figure 5:**
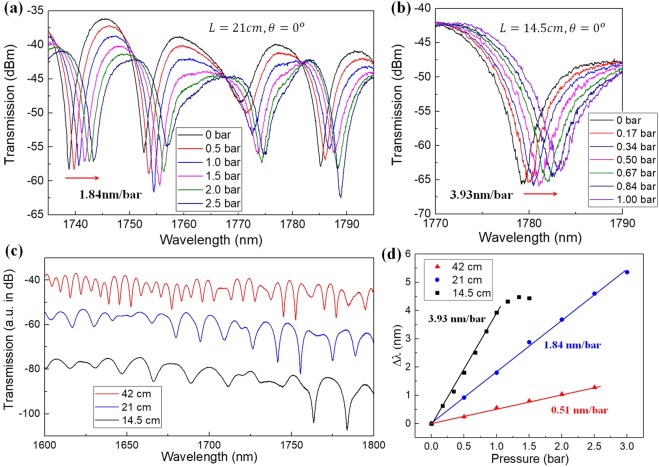
Transmission spectral evolution of a (**a**) 21 *cm* fiber and (**b**) 14.5 *cm* fiber under increasing gas pressure; (**c**) Transmission spectra of a DHAF interferometer at different lengths; (**d**) Performance of DHAF interferometric sensor. Shorter fiber offers higher sensitivity.

Figure 2(b) suggests a higher sensitivity with a shorter DHAF interferometer. In attempts to enhance the sensitivity, we tested a few different fiber lengths. As shown in Fig. 5(a,b), a shorter fiber interferometer indeed improves the sensor performance. We select a 1750 *nm* fringe dip instead of 1550 *nm* to avoid reduced visibility in a shorter wavelength, caused by increasing higher-order supermode contents (see Fig. 5(c)). The linear shift of the fringe dip around 1750 *nm* gives rise to 0.51, 1.84 and 3.93 *nm*/*bar* of sensitivity when the fiber gets shorten from 42 to 21 and 14.5 *cm*. The achieved sensitivity of 3.93 *nm*/*bar*, i.e., 39.3 *nm*/*MPa*, is 4 times larger than the state-of-the-art fiber sensors based on Mach-Zehnder interferometer^42^.

With the set OSA resolution of 0.04 *nm*, its minimum detectable pressure change becomes as low as 1.0 *kPa*. We note that the detectable range is highly dependent of the employed elastic material properties. The measured linear dependence of the interference phase change around 1750 *nm* is plotted in Fig. 5(d), demonstrating the super-sensitive performance of our hollow core fiber interferometric sensor.

**Figure 6:**
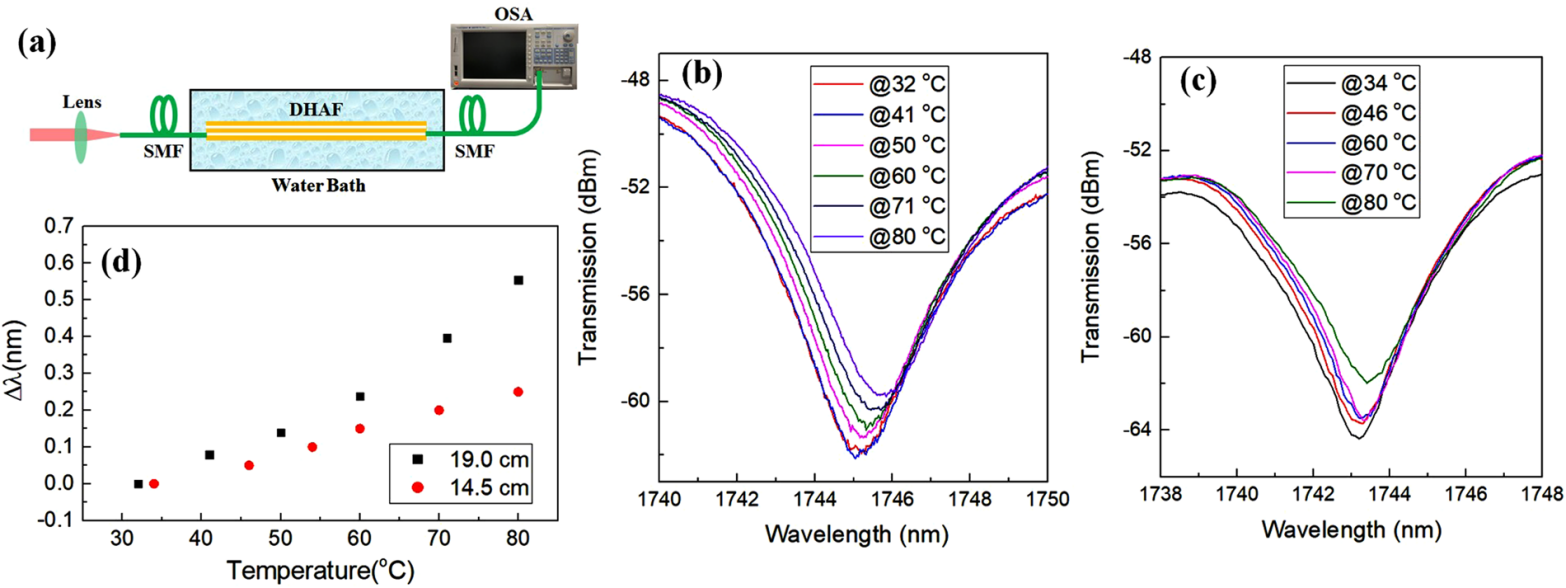
(**a**) Schematic of experiment setup for temperature sensitivity measurement. SMF: single mode fiber, OSA: optical spectrum analyzer; Red shift of interference fringes of (**b**) a 19 *cm* DHAF and (**c**) a 14.5 *cm* DHAF with increasing temperature; (**d**) Temperature induced wavelength shift of the fringe dip of the 19 *cm* and the 14.5 *cm* DHAFs.

Finally, we investigate temperature sensitivity of the interferometer. It is highly anticipated that the air core guidance suppresses the unwanted temperature sensitivity. To measure the temperature influence, two DHAF interferometers with different lengths (19 *cm* and 14.5 *cm*, respectively) are placed in a water bath as illustrated in Fig. 6(a). The temperature of the water bath gradually increases from room temperature to 80 °C with a step of around 10 °C. We can notice a small red shift of the interference fringe with increasing temperature as presented in both Fig. 6(b,c). It appears the dependence becomes less noticeable as fiber length gets shorter. As compared in Fig. 6(d), the average temperature sensitivity is reduced from 11.5 *pm*/°C to 5.4 *pm/*°*C* as the DHAF used is shortened from 19 *cm* to 14.5 *cm*. As the gas pressure sensitivity of a 14.5 *cm* DHAF interferometer is 39.3 *nm*/*MPa*, this contributes to 0.14 *kPa*/°*C* of temperature-pressure crosstalk, which is an order of magnitude improvement as compared to the state-of-the-art Mach-Zehnder interferometer based gas pressure sensor offering only 4.4 *kPa*/°*C* cross-talk with 43 *pm*/°*C* temperature sensitivity^42^. Hence, the DHAF can serve as an excellent gas pressure sensor in temperature varying atmosphere.

## Discussion

In summary, we investigate the opportunity of advancing anti-resonant fiber technology to a fiber device level. A dual hollow-core anti-resonant fiber is prepared as a platform of fiber interferometer. The two cores in the DHAF support supermodal interaction and generate spectral fringes as a basis for interferometric devices. The DHAF interferometer shows linear and ultra sensitive response with an additional unique feature of bending orientation dependent characteristic. This can offer additional dimension of interrogation for strain sensing applications. It is also demonstrated that sensitivity of the DHAF interferometer is adjustable by controlling the fiber length. The DHAF is polarization independent as well^6^.

The DHAF interferometer is further investigated as a gas sensor. In the past, fiber optics have made a meaningful progress in gas pressure sensing. Among various types of fiber sensors, a fiber modal interferometer, becomes a popular choice thanks to their high sensitivity. For instance, a several *nm* per *MPa* sensitivity has been reported using a Fabry-Pérot interferometer (FPI)^43^, but the temperature pressure cross-sensitivity remains as an open question. In addition, constructing FPIs in a fiber platform requires micro-processing in a fiber, which is not straightforward to implement in many cases and also compromises mechanical strength. On the other hand, the fiber sensors can make use of fiber Bragg grating (FBG), with a typical sensitivity in a range of tens to several hundreds of *pm* per *MPa*^44–47^. The limitations of the FBG sensors mainly relate to a small measurement range, and they are hardly usable in harsh environments^44^. In addition, the temperature pressure cross-sensitivity is a persisting issue in FBG based sensors. Alternatively, long period fiber grating (LPFG) has been employed to construct gas pressure sensors. Prior studies reported 51 *pm*/*MPa* in a Corning SMF-28 fiber^48^, 112 *pm*/*MPa* in a tapered PCF^49^, and 1.68 *nm*/*MPa* in an inflated PCF^50^, with a low cross-sensitivity of 1.8 *kPa*/°*C*. In comparison, a state-of-the-art Mach-Zehnder interferometer reported 9.6 *nm*/*MPa* sensitivity with a cross sensitivity of 4.4 *kPa*/°*C*^42^. Our DHAF interferometer shows much improved performance with 39.3 *nm*/*MPa* sensitivity and 0.14 *kPa*/°*C* low temperature cross-sensitivity. Compared to the state-of-the-art Mach-Zehnder interferometer based gas pressure sensor^42^, our DHAF interferometer offers 4 times larger sensitivity of 39.3 *nm*/*MPa*, and an order of magnitude improvement in the cross sensitivity. The successful demonstration confirms the applicability and the superiority of DHAF interferometer in sensing mechanical forces, thus contributing to advances in anti-resonant fiber technology and fiber sensing technology.

## Methods

### Multiple hollow-core fiber fabrication

F 300 tubes (16 *mm* × 20 *mm* of inner and outer diameters) and HSQ 300 rods (12*mm* outer diameter) from Heraeus were drawn into smaller capillaries and rods, respectively. The drawn capillaries and rods were then stacked together in a stacking rig that semi-automatically stacks the rods and capillaries to a desired structure. The stack was subsequently jacketed by another F 300 tube to form a preform in a diameter of 20 *mm*. A typical usable preform length was 10 *cm* which was pulled into several 1 *m* long cane with 2.5 *cm* diameter. Each cane could produce more than 10 *m* long fiber with relatively uniform structure. The detailed parameters of the cross-sectional structure of the fiber are given in Fig. S1(a). The images of a cane and a fiber are compared in Fig. 1(a,b), respectively. Within an 80 cm long fiber (which amounts to more than 2 device length), we examined the structure uniformity at every 20 *cm* interval under microscope. The structure showed ∼8% of variation in the air gap size, which did not affect the performance.

### Measurement of the gas pressure sensitivity

The experimental setup to measure the transmission spectrum of the DHAF interferometer under gas pressurization is illustrated in Fig. 4(a). A piece of DHAF was attached to an elastic material which was mounted in a custom-built gas pressure chamber. When the chamber is pressurized, the elastic material undergoes longitudinal distortion which results in stretching of the DHAF. A photograph of a pressure chamber is shown in Fig. S6 in the supplementary document. Light from a supercontinuum laser source (SC400 from Fianium) was then coupled into the DHAF using a plane-convex lens with 25 *mm* focal length and matching numerical aperture. The spot size at the focal point was small enough that the light could be precisely launched into an intended core (Core 1 or Core 2 only) of the DHAF (please, see Fig. S7 in the supplementary document for more details). A coupling efficiency was consistently maintained during the experiments. To measure the output signal from the DHAF, a single mode fiber (SMF28) was precisely aligned to a launched core of the DHAF, and subsequently spliced using an end view feature of an employed splicer (LZM-100, Fujikura) as presented in Fig. S8. The output signal was delivered to an OSA for spectrum measurement. In the measurement of the interference phase change under different gas pressures, the same result was obtained over 3 times at each gas pressure, which confirms its repeatability.

### Measurement of the temperature sensitivity

Figure 6(a) depicts our schematic to measure the temperature sensitivity of the DHAF interferometer. Each side of the DHAF was spliced with a SMF for signal input and signal output. At the input port, the laser beam from a supercontinuum source (SC400 from Fianium) was coupled into the SMF using a plane-convex lens with 6.2 *mm* focal length. At the output port, the SMF was connected to an OSA to measure the optical spectrum. A water bath (ALD-40500-150H, ALSTRON) was used to control temperature between 30 to 80 °C. The DHAF was fixed on the bottom of the water bath.

## Supplementary information

Supplementary Information
