# Supplementary material for: Multiple hollow-core anti-resonant fiber as a supermodal fiber interferometer
Source: Sci Rep. 2019 Jun 27;9:9342. doi: 10.1038/s41598-019-45771-2 (PMC6597538; doi:10.1038/s41598-019-45771-2)
Supplement: Supplementary file 1 — Supplementary Information [file 41598_2019_45771_MOESM1_ESM.pdf]

Multiple hollow-core anti-resonant fiber as a supermodal fiber interferometer

Xiaosheng Huang[1] , Jichao Zang[1], and Seongwoo Yoo[1,*]

[1] School of Electrical and Electronics Engineering, The Photonics Institute, Nanyang Technological University, Singapore 639798, Singapore
*seon.yoo@ntu.edu.sg

1. Supermodes in a DHAF

Supermodes in the DHAF are simulated with Comsol Multiphysics. The DHAF has a wall thickness, t, of 1.21 µm, a gap width, g, of 9.4 µm, a capillary diameter, p, of 19.0 µm, and a core diameter, D_i, of 28.0 µm, where i is 1 or 2 as illustrated in Fig. S1(a). Effective refractive indices of the low order modes are calculated for x and y polarization. As shown in Fig. S2, Δn_{eff} between the first and second order supermodes is around 8.4×10^{-4} at 1650 nm. According to Eq. (1) in the manuscript, when L=38 cm, its corresponding free spectral range around 1650 nm is calculated to be 8.5 nm, which matches with the measured value of 8.2 nm as shown in Fig. 1(b). Therefore, the fast oscillatory pattern originates from the interference between first order and second order supermodes. This conclusion is also supported by the near field mode image in Fig. S1(b), which indicates presence of the first and the second order supermodes. On the contrary, the slow varying envelope in Fig. 1(b) in the manuscript originates from the interference between symmetric and anti-symmetric supermodes with the same order, whose Δn_{eff} is in a level of 10^{-6}. According to Fig. S2, the Δn_{eff} between x-polarized and y-polarized supermodes is negligible, thus indicating low birefringence. The low birefringence of DHAF is due to the low energy overlap between airy core mode and capillary walls[s1,s2].

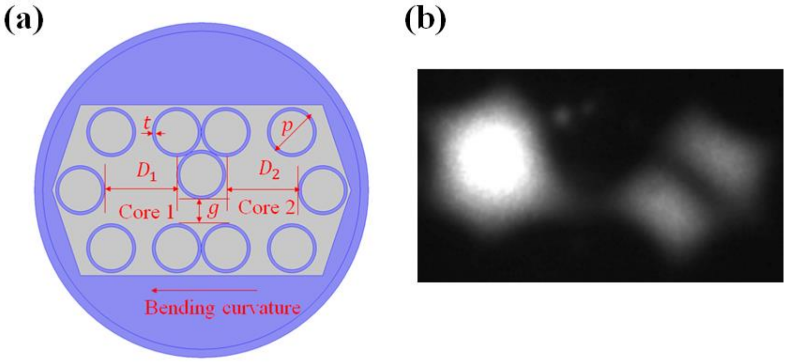

Figure S1. (a) Geometry of the structure used for simulation, t=1.21 µm, g=9.4 µm, p=19.0 µm, when the fiber is kept straight, D_1=D_2=28.0 µm. (b) Near field mode image when light is coupled into Core 1 of a 38 cm DHAF.

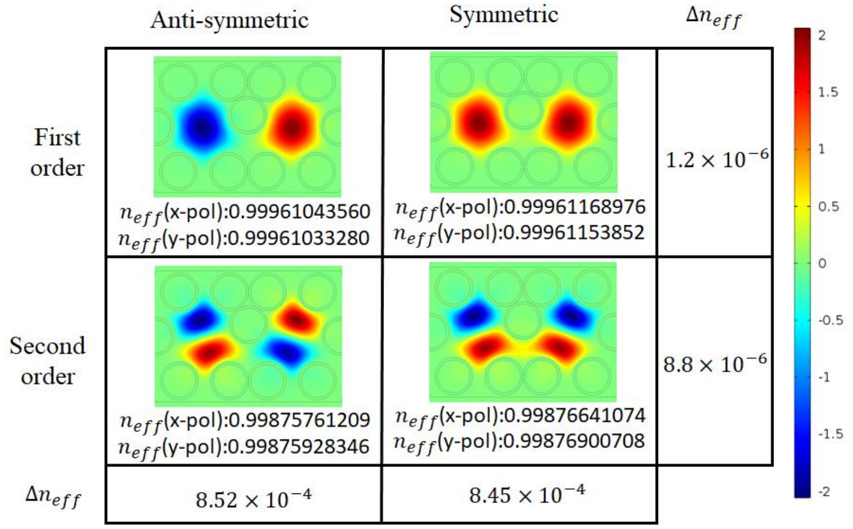

Figure S2. Lower order supermodes in the DHAF as shown in Fig. S1(a), Δn_{eff} in row represents the index difference between the first and second order modes, while Δn_{eff} in column represents the index difference between symmetric and anti-symmetric modes.

2. Bending direction dependent wavelength shift

Figure 2(b) in the manuscript presents a bending orientation dependent sensitivity of the fringe dips. To distinguish the direction angle of the DHAF in the experiment, we firstly make sure the fiber is straight without any twist on the stages, and then we verify that the arrangement of the two cores is aligned at both ends using a camera. When the fiber is bent at 0°, Core 1 is located below Core 2 as illustrated in Fig. S3. To achieve 180°, the fiber is rotated half turn so that Core 1 is located above Core 2. Furthermore, when the angle becomes 90°, the transverse axis is out of the bending plane. We postulate that the dependence accounts for structural distortion under bending. As shown in Fig. S3, when bending is applied at θ = 0°, Core 1 is located in the outer curvature, and subject

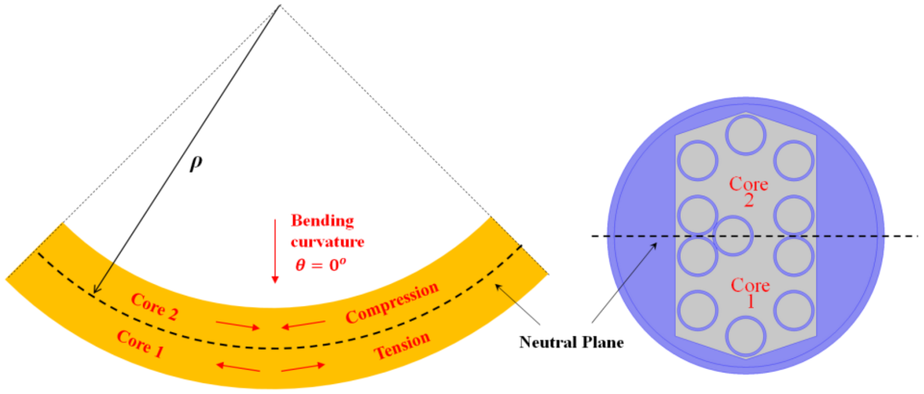

Figure S3. Stress distribution within a DHAF under bending at θ = 0° (see Fig. 2 in the manuscript for the definition of θ).

to tensile stress. The tensile stress effectively reduces core size. In contrast, Core 2 is placed in the inner curvature, thus being under compression stress, leading to effectively large core[s3,s4]. Hence, the bending can introduce asymmetric core sizes.

The axial bending strain, ε, can be estimated from the following formula[s5]:

$$\varepsilon = \frac{y}{\rho} \tag{S1}$$

where ρ is the radius of the bending curvature, and y is the distance from the core boundary to the neutral plane. The neutral plane is a plane in which no excess longitudinal stress or strain is applied. The applied longitudinal strain by bending can influence the core size, which can be evaluated from the effective index change of the fundamental mode, Δn_{eff} (FM). As indicated in Fig. 5 in Ref. [S6], Δn_{eff} (FM) is proportional to ε, and $\varepsilon = 0.01$ corresponds to Δn_{eff} (FM) $= -2 \times 10^{-4}$. As shown in Fig. S4, the bending radius is 90 cm when a 38 cm fiber undergoes 50 μm compression. According to Eq. (S1), Core 1 becomes subject to a longitudinal strain of $\varepsilon = 4.2 \times 10^{-5}$, resulting in Δn_{eff} (FM) $= -8.4 \times 10^{-7}$. This corresponds to 0.16 % variation of core size due to longitudinal strain. As the original core size was measured as 28.0 μm in a straight fiber, we set core sizes under bending as $D_1 = 27.955$ μm, $D_2 = 28.045$ μm.

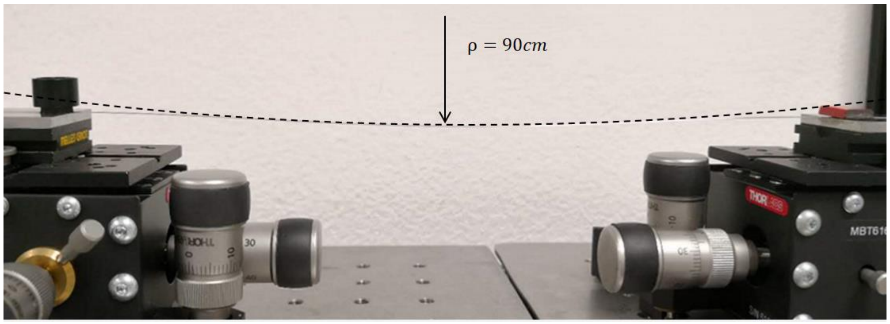

Figure S4. Applying 50 μm compression to a 38 cm fiber introduces a 90 cm radius bending curvature.

The simulation results are represented in Fig. S5. Since the DHAF has low birefringence, only x-polarized supermodes are shown for brevity. Under the bending at $\theta = 0^{\circ}$, Core 1 contains strong anti-symmetric supermodes whereas Core 2 sees mainly the symmetric supermodes[s7]. Compared with straight fiber in Fig. S2, the Δn_{eff} in Core 1 increases by 0.13 % leading to a 2.1 nm red shift of fringe while Core 2 experiences decreased Δn_{eff} by 0.08 % resulting in a 1.3 nm blue shift. This well explains the experimental bending orientation dependent sensitivity of the DHAF interferometer.

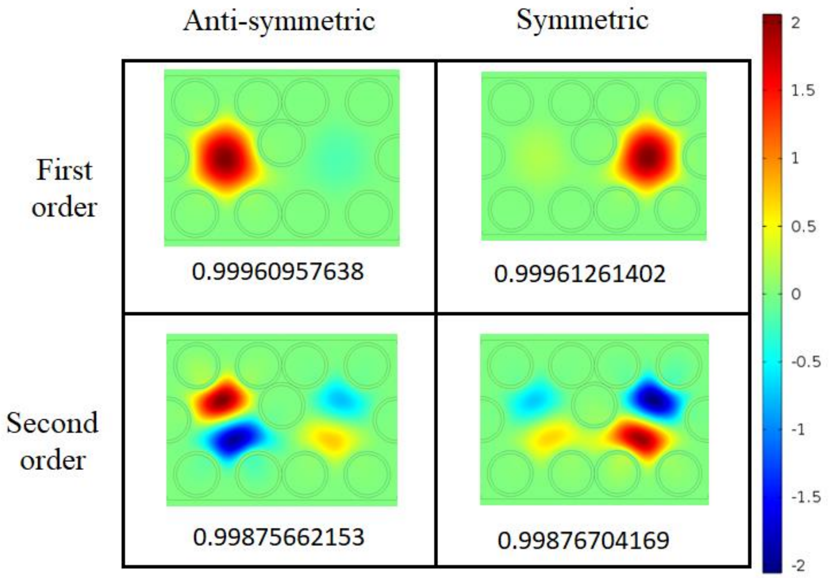

Figure S5. Simulated supermodes (x-polarized) and their effective refractive indices in a DHAF when it is bent at θ = 0°.

3. Gas pressure chamber

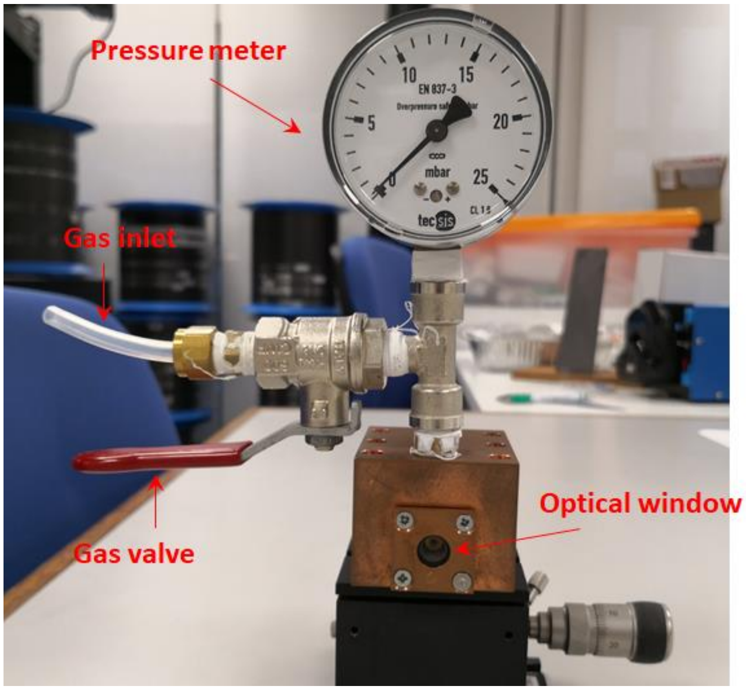

Figure S6. Photograph of the chamber used for introducing gas pressure. The chamber is equipped with an optical window and can be mounted on a translational stage.

4. Evidence of light coupled into a single core

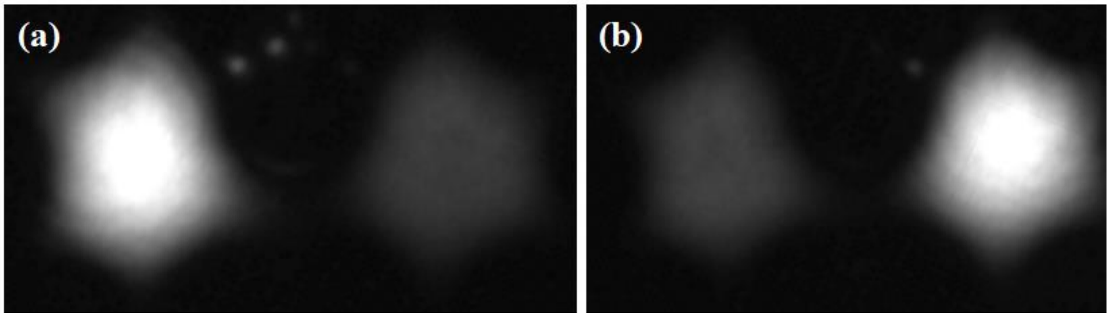

Figure S7. Near field mode image of a 10 cm DHAF when (a) light is launched into the left core; (b) the fiber is stretched by around 40 μm (equals to the core separation distance) and the light is coupled into the right core.

5. Aligning and Splicing SMF with DHAF

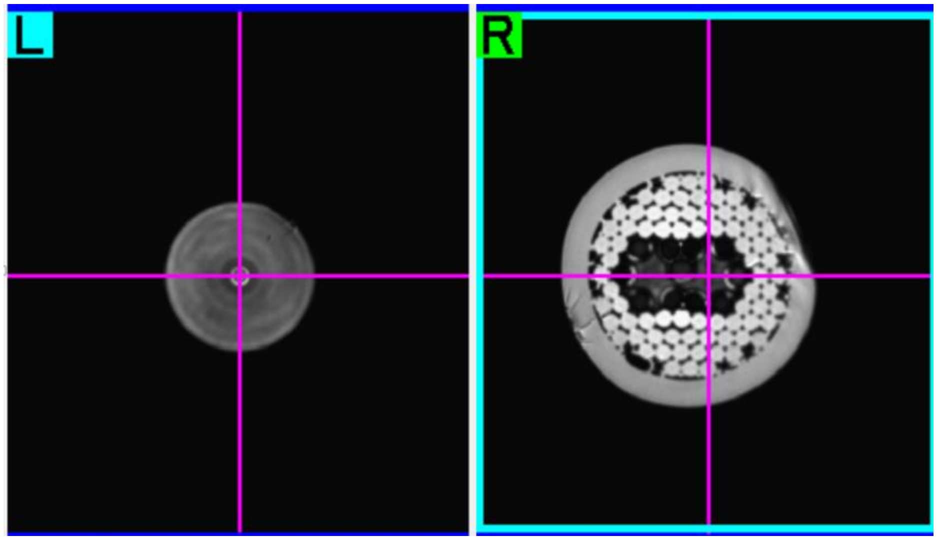

Figure S8. End view operation window of LZM-100 (Fujikura, Japan). User can manually control the location of the magenta lines, and then the splicer will move the cross point of the magenta lines to the center. In this way, the core of the SMF (left) and the DHAF (right) can be matched.

References

[S1] Ding, W. & Wang, Y.-Y. Hybrid transmission bands and large birefringence in hollow-core anti-resonant fibers. *Opt. Express* **23**, 21165–21174, DOI: https://doi.org/10.1364/OE.23.021165 (2015).

[S2] Mousavi, S. A., Sandoghchi, S. R., Richardson, D. J. & Poletti, F. Broadband high birefringence and polarizing hollow core antiresonant fibers. *Opt. Express* **24**, 22943–22958, DOI: https://doi.org/10.1364/OE.24.022943 (2016).

[S3] MacPherson, W., Gander, M. R., MJ, Jones, B. P., JDC, Burnett, G. A., JG & Mangan, B. T. K.-J. R. P., B. Remotely addressed optical fibre curvature sensor using multicore photonic

crystal fibre. *Opt. Commun.* **193**, 97–104, DOI: https://doi.org/10.1016/S0030-4018(01)01260-3 (2001).

[S4] Wikipedia, "Bending," https://en.wikipedia.org/wiki/Bending.

[S5] Gramoll, K. A web-based electronic book (ebook) for solid mechanics. *In Proc. ASEE Annual Conf.(Mech. Div. Best Pres.Award), Honolulu, HI, USA* (Citeseer, 2007).

[S6] Pang, M., Xuan, H., Ju, J. & Jin, W. Influence of strain and pressure to the effective refractive index of the fundamental mode of hollow-core photonic bandgap fibers. *Opt. Express* **18**, 14041–14055, DOI: https://doi.org/10.1364/OE.18.014041 (2010).

[S7] Kumar, N., Shenoy, M., Thyagarajan, K. & Pal, B. Graphical representation of the supermode theory of a waveguide directional coupler. *Fiber integrated optics* **25**, 231–244, DOI: https://www.tandfonline.com/doi/abs/10.1080/01468030600569925 (2006).
